# Meteorological, Socioeconomic, and Environmental Factors Influencing Human Brucellosis Occurrence in Yunnan, China, 2006–2021: A Bayesian Spatiotemporal Modeling Study

**DOI:** 10.1155/tbed/8872434

**Published:** 2025-07-16

**Authors:** Ke Li, Jidan Zhang, Binbin Yu, Michael P. Ward, Mengxin Liu, Yuanhua Liu, Zengliang Wang, Zhuohang Chen, Wenjin Li, Na Wang, Yu Zhao, Xiangdong Yang, Fuping Yang, Peng Wang, Zhijie Zhang

**Affiliations:** ^1^Shanghai Institute of Infectious Disease and Biosecurity, Fudan University, Shanghai, China; ^2^Department of Epidemiology and Health Statistics, School of Public Health, Fudan University, Shanghai, China; ^3^Key Laboratory of Public Health Safety, Ministry of Education, Shanghai, China; ^4^Zoonosis Control and Prevention Department, Yunnan Institute of Endemic Diseases Control and Prevention, Kunming, Yunnan, China; ^5^Sydney School of Veterinary Science, The University of Sydney, Sydney, New South Wales, Australia; ^6^Department of Epidemiology, School of Public Health, Cheeloo College of Medicine, Shandong University, Jinan, Shandong, China

**Keywords:** Bayesian spatiotemporal model, brucellosis, influencing factors, zoonosis

## Abstract

**Background:** Brucellosis epidemics in Yunnan Province in southern China have increased and caused more impact in recent years. However, the epidemiological characteristics and driving factors for brucellosis have not been clearly described. The aim of this study was to analyze the spatiotemporal distribution and potential factors for human brucellosis (HB) in Yunnan Province, 2006–2021.

**Methods:** HB data were obtained from the China National Notifiable Infectious Diseases Reporting Information System. Global spatial autocorrelation and spatial scanning statistics were used to analyze the spatial patterns of brucellosis. Zero-inflated negative binomial (ZINB) Bayesian spatiotemporal models were applied to the analysis of potential risk factors, including environmental, meteorological, and socioeconomic factors.

**Findings:** Between 2006 and 2021, a total of 2794 brucellosis cases were reported. The central and western regions were the most severely affected. GDP showed a positive correlation with brucellosis risk when in the range 0–30.9 billion RMB, peaking with a relative risk (RR) of 13.64 (95% Bayesian credible interval [BCI]: 4.10, 49.10) at around 2.3 billion RMB. Conversely, a negative correlation was observed for GDP between 101 and 135 billion RMB, with the RR dropping to 0.14 (95% BCI: 0.01, 0.89) at 135 billion RMB. Brucellosis cases increased by 4.90% (95% BCI: 1.82%, 7.95%) per 1°C increase in temperature, while a 1° increase in slope reduced cases by 17.06% (95% BCI: 4.01%, 28.81%).

**Interpretation:** Our findings suggest that socioeconomic factors play the greatest role in the occurrence of brucellosis in both northern and southern China; however, the effects of the environmental factors may be different between these areas. Differences in factors affecting each region need to be fully considered, and brucellosis prevention and control need to be adapted to these differences.

## 1. Introduction

Brucellosis is an important zoonotic disease caused by *Brucella* species [1]. There are reports of human brucellosis (HB) cases from at least 170 countries, and the World Health Organization continues to classify it as a neglected zoonotic disease. Globally, it is estimated that between 1.6 and 2.1 million new HB cases occur each year [2]. The primary modes of transmission are the ingestion of contaminated food products, such as milk and other dairy products, and direct contact with infected animals [3]. Livestock, particularly sheep, goats, and cattle, serve as the primary reservoirs of *Brucella* bacteria [4]. In animals, brucellosis causes reproductive complications, such as abortions, stillbirths, and decreased milk yield. In humans, it manifests with symptoms like fever, night sweats, myalgia, and arthralgia, leading to profound consequences for both public health and agricultural productivity [1, 5].

HB remains a major public health concern in China. Since the implementation of passive surveillance in 2004, the number of annually reported cases has generally increased, with a peak of 67,616 confirmed cases in 2023. Current brucellosis control strategies in China include disease surveillance, culling of infected animals, and animal vaccination, although vaccination efforts are limited to high-incidence areas [6]. Historically, brucellosis has been more prevalent in northern China, where a gradual decline in case numbers has been observed. In contrast, the disease is increasingly reported in southern China, with Yunnan Province exhibiting the most significant increase [7, 8].

Identifying the factors contributing to HB incidence is essential to explain the worsening epidemic and ongoing spread in Yunnan [9]. Regarding economic factors, Peng et al. found an association between GDP, the number of hospital beds, and brucellosis cases [10, 11]. Numerous studies have identified livestock-related factors, including the number of cattle and sheep, the number of livestock enterprises, and the consumption of milk and meat, as being associated with brucellosis [1, 4, 12]. With respect to meteorological factors, previous studies have shown significant associations between HB and variables such as precipitation, sunshine duration, wind speed, temperature, and relative humidity [11, 13]. Additionally, several studies have reported that environmental factors such as nighttime lighting, land cover, altitude, and normalized difference vegetation index (NDVI) are also associated with the development of HB [1, 12]. However, these factors differ between northern and southern China, potentially leading to different epidemic patterns and transmission dynamics. Previous studies investigating the factors that influence HB have predominantly focused on high-prevalence regions in northern China, and their findings might not be directly applicable to Yunnan Province. Furthermore, the variables assessed in these studies are not consistent, introducing potential bias, and the findings for the same variables have often shown considerable heterogeneity.

In this study, we investigated the spatial and temporal patterns of HB and used Bayesian spatiotemporal models to comprehensively assess the influence of meteorological, socioeconomic, and environmental factors on the incidence of HB in Yunnan Province, southern China.

## 2. Methods

### 2.1. Study Area

**Figure 1:**
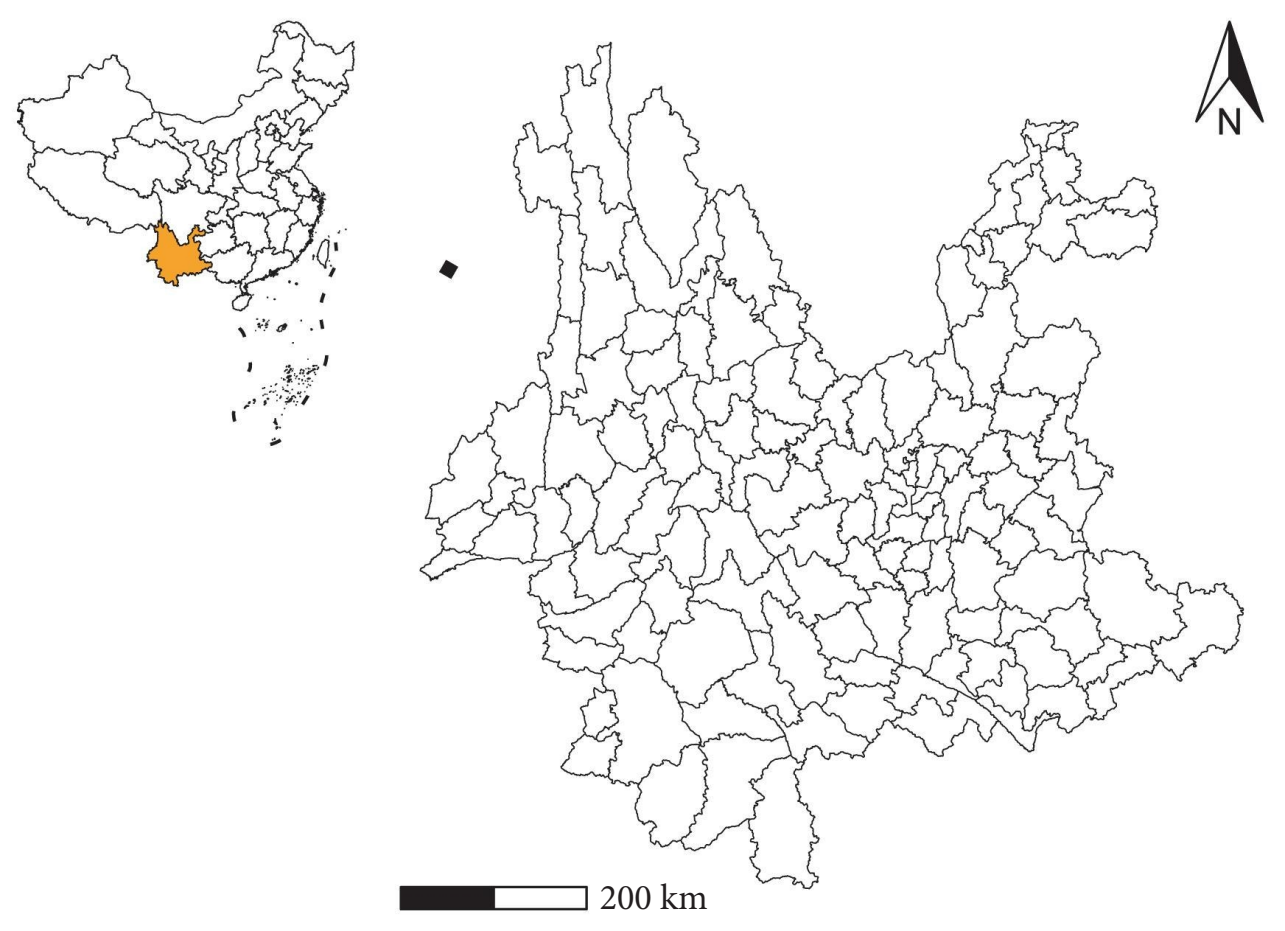
Location of the study area and county-level administrative map of Yunnan Province. Map data source: National Platform for Common Geospatial Information Services (https://cloudcenter.tianditu.gov.cn/administrativeDivision/).

Yunnan Province is situated in southwestern China (21°8'–29°15′ N, 97°31′–106°11′ E), sharing international borders with Myanmar, Laos, and Vietnam. The province covers an area of 394,100 km^2^, had a total population of 46.93 million at the end of 2022, and is subdivided into 16 municipalities, further divided into 129 counties (Figure 1). In 2024, Yunnan Province ranked second nationwide in both goat inventory and beef cattle slaughter [14].

### 2.2. Data Resources

#### 2.2.1. HB Data

HB cases in Yunnan Province from 2006 to 2021 were obtained from the National Notifiable Infectious Diseases Reporting Information System, an internal database maintained by the China CDC. Yunnan Institute of Endemic Diseases Control and Prevention holds access rights. HB cases were strictly confirmed based on national diagnostic protocols in China (Diagnostic Criteria for Brucellosis, WS 269–2007/WS 269–2019), which incorporate epidemiologic exposure history (e.g., contact with infected animals or contaminated animal products or residence in endemic areas), clinical manifestations (e.g., undulating fever, headaches, myalgia, arthralgia, fatigue, and sweats), and laboratory test results (including primary screening and confirmatory tests) [12].

#### 2.2.2. Environmental Data

The environmental factors analyzed included NDVI, land cover, distance to the nearest water body, water body area, slope, and elevation. NDVI data were obtained from the Level 1 and Atmosphere Archive and Distribution System (https://ladsweb.modaps.eosdis.nasa.gov/) using the monthly global 1-km resolution product. Data preprocessing included the removal of negative values, and then maximum values were synthesized and aggregated at the county scales. Land cover data were obtained from the Landsat-derived annual land cover product of China [15]. The number of greened cells in each county as a percentage of all image cells was then calculated to create a new variable, “Green coverage.” Elevation and slope data were extracted from the digital elevation model (DEM) provided by the Shuttle Radar Topography Mission (SRTM). Mean elevation and slope were calculated at the county level.

#### 2.2.3. Socioeconomic Data

The socioeconomic factors analyzed included GDP, population, and nightlight. County-level GDP and population data were obtained from the Statistical Yearbook of Yunnan Province Bureau of Statistics [16]. Nightlight data, which serve as a proxy for human activity and urban prosperity, were obtained from the National Earth System Science Data Center (https://www.geodata.cn), with a temporal resolution of 1 year and a spatial resolution of 500 m [17]. The nightlight for each county was calculated as the average brightness of all cells within the county boundary.

#### 2.2.4. Meteorological Data

Meteorological factors analyzed included temperature, precipitation, and wind speed. All data were retrieved from the China Meteorological Data Sharing Service System (http://data.cma.cn/). The nationwide daily meteorological data were obtained from 602 meteorological stations. Kriging was applied to interpolate the point data and create 1 km × 1 km gridded meteorological surface data, which were then aggregated at monthly and county levels [18].

### 2.3. Statistical Analysis

#### 2.3.1. Spatial Autocorrelation and Spatial Cluster Analysis

Given the small number of cases in certain years, data were aggregated into 5-year intervals for analysis. Spatial autocorrelation analysis was assessed using Global Moran's *I* to evaluate whether brucellosis incidence exhibited spatial dependence [19]. Retrospective spatial scan statistics based on a Poisson distribution model (cases per population at risk) were used to detect high-risk areas. The log-likelihood ratio (LLR), corresponding *p*-values, and relative risk (RR) were calculated for scanning windows of various sizes, with the maximum scan radius set at 10% of the population at risk, and 999 Monte Carlo simulations were performed. For *p* < 0.05, a higher LLR indicated a greater likelihood of brucellosis clustering within the scanning window, suggesting that the window represented a statistically significant cluster [20].

#### 2.3.2. Bayesian Spatiotemporal Model

First, to avoid collinearity, Pearson correlation analysis was conducted to assess the correlation between candidate covariates, retaining only those with a correlation coefficient of less than 0.6 [21]. Variables that were not statistically significant were excluded through univariate Bayesian modeling [22]. Linear and nonlinear representation of variables was determined by the use of a generalized additive model [23].

Second, given the excess zeros count observed in counties with low brucellosis incidence, a zero-inflated negative binomial (ZINB) model was employed to account for overdispersion and better capture the data's underlying structure. Four Bayesian models were developed, each incorporating independent variables and different spatiotemporal effect terms. Model 1 contained structured and unstructured temporal effects; Model 2 contained structured and unstructured spatial effects; Model 3 contained all of the above; and Model 4 extends Model 3 by adding spatiotemporal interaction effects. Model performance was evaluated using the deviance information criterion (DIC) and the Watanabe–Akaike information criterion (WAIC). In the models, brucellosis cases (*y*_st_) were modeled using a ZINB distribution. The expected number of cases was modeled based on monthly data (*t* = 1–228) over 19 years for each county (*s* = 1–129), with the logarithm of the population (*P*_st_) included as an offset.

(1) $$\begin{array}{c} y_{\text{st}}∼\text{ZINB}\left(\mu_{\text{st}},\kappa \right), \end{array}$$

(2) $$\begin{array}{c} \log\left(\mu_{\text{st}}\right)=\log\left(P_{\text{st}}\right)+\log\left(\rho_{\text{st}}\right), \end{array}$$

(3) $$\begin{array}{c} \log\left(\rho_{\text{st}}\right)=\alpha +\sum \beta_{i}x_{\text{ist}}+\gamma_{t}+\mu_{t}+\nu_{s}+\delta_{s}+\varphi_{\text{st}}. \end{array}$$

Spatial effects were specified using the Besag–York–Mollié (BYM) model, which combines an intrinsic conditional autoregressive model for spatially structured effects *ν*_*s*_, and an independent identically distributed Gaussian model for spatially unstructured effects *δ*_*s*_. Temporal structured effects (*γ*_*t*_) were modeled using a first-order random walk latent process, assuming that brucellosis incidence in 1 month depends on the incidence in the previous month. Spatiotemporal interaction effect (*φ*_st_) was represented using two unstructured components and were treated as unobserved covariates. The equations are given and described in the Supporting Information (pp 2).

For all analyses, a significance level (*α*) of 0.05 was used, with statistical significance indicated by 95% Bayesian credible interval (BCI) for RRs that excluded 1. Spatial scan statistics were conducted using SaTScan (version 10.0.2; Cambridge, MA, USA), while all other analyses were performed in R (version 4.4.1; R Core Team, Vienna, Austria). The *terra* package was used for raster data, *sf* for vector data, and *INLA* for Bayesian spatiotemporal modeling.

## 3. Results

**Figure 2:**
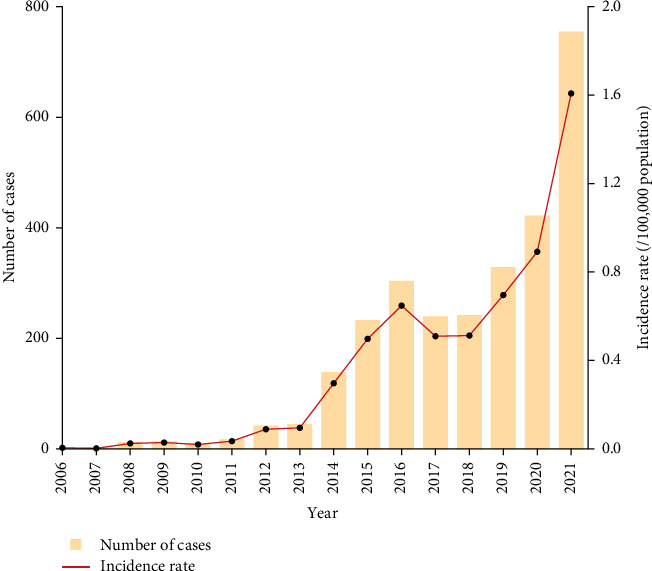
Reported human brucellosis cases (*N* = 2794) in Yunnan Province, 2006–2021. Annual total number of cases (orange bars) and incidence rate per 100,000 population (red line).

**Figure 3:**
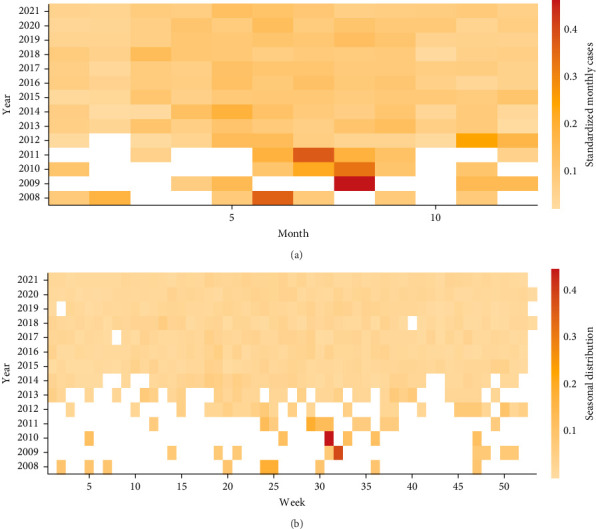
Heatmap of human brucellosis cases in Yunnan Province, 2006–2021. (A) Monthly time series of cases from 2006 to 2021, standardized by the annual number of cases. (B) Weekly time series of cases from 2006 to 2021, standardized by the annual number of cases.

**Figure 4:**
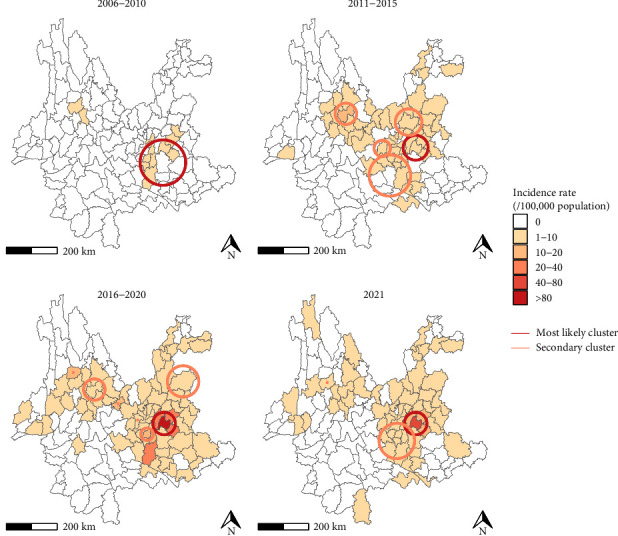
Geographic distribution of the annual incidence rate (per 100,000 population) and spatial scanning analysis results of human brucellosis in Yunnan Province. Shades of color indicate prevalence, and circles indicate the location of spatial aggregation areas.

Between 2006 and 2021, a total of 2794 brucellosis cases were reported. The male-to-female sex ratio was about 2.3:1. The median age of patients was 49 years, with the highest number of cases occurring in the 50–59 age group. Individuals aged 30–69 accounted for 81.5% of all cases. Farmers represented the majority of patients (82.6%), followed by students and children (5.9%) (Table 1). The incidence of brucellosis in Yunnan showed an overall increasing trend, with two peaks: 2016 (303 cases, 0.65 cases per 100,000 population) and 2021 (754 cases, 1.61 cases per 100,000 population) (Figure 2). Furthermore, 68.47% (1913/2794) of cases occurred between March and September, though no clear seasonal pattern was observed at either the monthly and weekly scales after standardization (Figure 3). Cumulatively, during the study period cases of brucellosis were reported from 86 (66.7%) of the 129 counties in Yunnan Province. The epidemic expanded markedly over time, spreading outward from the central-western and northeastern regions. Between 2006 and 2021, the top three counties with the highest average annual incidence rates were Shilin Yi nationality Autonomous County (9.80 per 100,000 population), Luxi County (3.41 per 100,000 population), and Luliang County (2.78 per 100,000 population) (Figure 4).

The results of global autocorrelation analyses conducted across 5-year intervals indicated that the Moran's *I* values were consistently greater than 0 and gradually increased, with corresponding *p*-values of less than 0.01. These findings indicated significant global spatial clustering in the distribution of brucellosis in Yunnan (Table S1). Spatial scan statistics identified the primary clusters in the central and western regions of Yunnan. Shilin Yi Nationality Autonomous County, Luxi County, and Yiliang County were consistently included in the most likely clusters throughout the study period. Secondary clusters were predominantly concentrated in the central-western and northeastern regions of the province (Figure 4).

**Figure 5:**
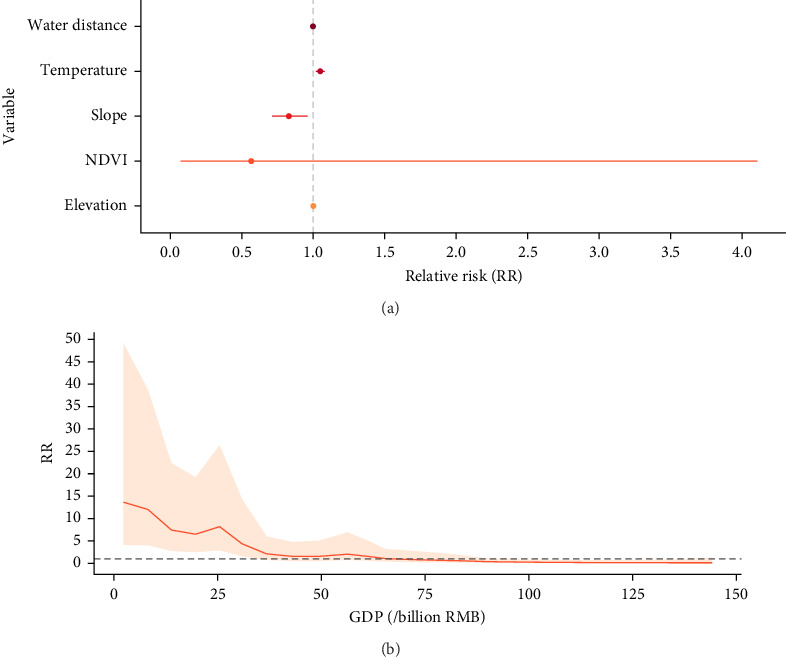
Factors influencing brucellosis based on the Bayesian spatiotemporal interaction model in Yunnan, 2006–2021. (A) Relative risks and 95% BCI for covariates with fixed effects in the model. (B) Nonlinear relationship between GDP and brucellosis in the model.

The descriptive characteristics of all variables are provided in Table S2. The results of variable selection and Bayesian spatiotemporal model comparison are provided in Figure S1, Tables S3 and S4. The optimal model is Model 4, which includes elevation, slope, NDVI, temperature, GDP (nonlinear), and the distance to the nearest water body as covariates. Notably, the nonlinear relationship between GDP and brucellosis shows a decreasing trend in RR as GDP increases. A positive correlation is observed when GDP is within the range 0–30.9 billion RMB, peaking at an RR of 13.64 (95% BCI: 4.10, 49.10) at around 2.3 billion RMB. In contrast, a negative correlation is observed when GDP is within the range of ~101–135 billion RMB, with the RR reaching its lowest value of 0.14 (95% BCI: 0.01, 0.89) at 135 billion RMB. Brucellosis cases increased by 4.90% (95% BCI: 1.82%, 7.95%) for each 1°C increase in temperature, while an increase in the slope of 1° was associated with a 17.06% (95% BCI: 4.01%, 28.81%,) reduction in cases (Figure 5).

## 4. Discussion

We described the county-level spatiotemporal distribution and dynamic patterns of HB and analyzed potential risk factors for brucellosis in Yunnan from 2006 to 2021. This study is the first of its kind in a southern province of China, with comprehensive consideration of potential meteorological, socioeconomic, and environmental factors. It is not only of great practical significance for addressing the brucellosis epidemic in Yunnan Province but also provides theoretical references for the prevention and control of brucellosis in newly infected areas in other southern provinces of China. It offers a scientific basis for the development of effective prevention and control strategies in the northern and southern provinces of China.

In this study, GDP showed opposite associations with brucellosis at lower and higher values. In counties in Yunnan with lower GDP, the implementation of poverty alleviation policies in recent years has significantly promoted livestock farming, contributing to increases in both GDP and brucellosis incidence. However, in areas with higher GDP, industry predominates, which might compete to some extent with the development of animal husbandry and thereby suppressing the increase of brucellosis. This finding aligns with Zhao et al. [12] on brucellosis in Xi'an using boosted regression tree analysis, which also revealed that brucellosis occurrence initially increased significantly with rising GDP and then stabilized. Meanwhile, we suggest that socioeconomic factors have a greater impact on the occurrence of HB than other factors, both in the south and in the north [12, 24].

The maximum temperature in Yunnan can reach 26°C. As the temperature rises, it approaches the optimal growth temperature for *Brucella*, leading to a higher risk of brucellosis [25, 26]. There is a significant contrast in the natural environmental factors between the north and south of China. In northern regions such as Inner Mongolia, plains dominate, with low elevations and few water bodies, while southern regions like Yunnan feature highland terrain with varied elevations and abundant water bodies. However, the confidence intervals for the RRs of the natural environmental factors, including NDVI, elevation, and the nearest distance to the water body, all include 1. One possibility is that the effects of socioeconomic factors are much stronger than those of these natural environmental factors. Another possibility is that natural environmental factors affect brucellosis incidence primarily by influencing human livestock activities (socioeconomic factors). Additionally, our results for elevation and NDVI differed from previous studies, and slope and distance to the water body were considered for the first time [1, 5, 26]. While we were unable to compare the results due to the lack of previous studies on natural environmental factors in the northern provinces, the results of the current study suggest that environmental factors might not be critical in the occurrence of HB in southern areas of China.

In this study, we did not observe a clear seasonal pattern in brucellosis incidence in Yunnan. This finding differs from previous studies conducted nationwide or in northern provinces but is consistent with the results of Lai et al. [27], who analyzed seasonal patterns in both northern and southern provinces using national data from 1955 to 2014 [7, 28]. We hypothesize that this difference may be related to regional variations in farming structure. Northern provinces—such as Inner Mongolia—are dominated by velvet goats, and the production of velvet and reproduction in summer and fall may lead to an increased risk of exposure and a higher number of cases [29]. In contrast, the heavily outbreak-affected areas of Yunnan mainly raise dairy goats that produce milk all year round and are predominantly free-range and hand-milked by farmers. This type of livestock management might result in a relatively stable risk of brucellosis transmission throughout the year [30].

Previous studies have consistently identified sheep stock numbers as the most critical factor influencing brucellosis [11, 31, 32]. Although we collected relevant data, it was not included in the model for two main reasons. First, according to local CDC investigations, brucellosis in Yunnan Province is primarily associated with dairy goats [33], while the sheep stock number data includes both sheep and goats (which in turn includes dairy and meat goats). In some areas of Yunnan, the scale of meat goat farming is exceptionally large, rendering the sheep stock number data either unusable or biased in terms of analysis of correlations with HB. Second, many counties in Yunnan have a dietary tradition of consuming mutton, milk, and dairy products, such as milk pastries. Therefore, processing might also be an important link in contributing to local outbreaks, in addition to farming.

There are several limitations to our study. The first limitation is that the passive surveillance system might underestimate cases due to latent infections, underreporting, and mildly symptomatic patients who do not actively seek medical attention. Additionally, the diagnostic capacity of healthcare workers has improved significantly in recent years through training by the relevant authorities in Yunnan Province, which might have contributed to the increase in the number of cases reported. Second, sheep stock could not be included in the model because it did not adequately represent the farming structure and potential infection links in Yunnan [33]. Finally, we included many natural environmental factors because they differed markedly between northern and southern China. However, due to the limitations of previous studies, we are unable to provide a comprehensive and insightful discussion with regard to the associations we identified.

## 5. Conclusions

Our findings suggest that GDP, temperature, and slope are associated with the incidence of brucellosis in Yunnan. Socioeconomic factors play the largest role in brucellosis incidence in both northern and southern provinces of China; however, environmental factors may affect different regions differently. There is a need to fully consider the differences in influencing factors among regions and to adjust brucellosis prevention and control measures according to these differences.

## Figures and Tables

**Table 1:** Demographic and diagnostic characteristics of patients with human brucellosis, categorized by diagnosis type, Yunnan Province, 2006–2021^a^.

Characteristic	Total *N* = 3809	Clinical diagnostic cases *N* = 471	Confirmed cases *N* = 3180	Probable cases *N* = 158
Sex				
M	2648 (69.5)	332 (70.5)	2197 (69.1)	119 (75.3)
F	1161 (30.5)	139 (29.5)	983 (30.9)	39 (24.7)
Median age, year, (IQR)	49 (38–57)	50 (39–57)	49 (38–57)	52 (40–61)
Age group, year				
0–9	118 (3.1)	15 (3.2)	101 (3.2)	2 (1.3)
10–19	122 (3.2)	13 (2.8)	105 (3.3)	4 (2.5)
20–29	303 (8.0)	41 (8.7)	252 (7.9)	10 (6.3)
30–39	504 (13.2)	56 (11.9)	425 (13.4)	23 (14.6)
40–49	867 (22.8)	106 (22.5)	729 (22.9)	32 (20.3)
50–59	1160 (30.5)	151 (32.1)	963 (30.3)	46 (29.1)
60–69	569 (14.9)	69 (14.6)	467 (14.7)	33 (20.9)
≥70	166 (4.4)	20 (4.2)	138 (4.3)	8 (5.1)
Occupation				
Farmer or herdsman	3253 (85.9)	400 (85.3)	2714 (85.9)	139 (87.9)
Infants or students^b^	213 (5.6)	27 (5.8)	180 (5.7)	6 (3.8)
Others	322 (8.5)	42 (9.0)	267 (8.4)	13 (8.2)
Median onset to diagnosis interval, day (IQR)	12.0 (6.0, 31.0)	10.0 (4.0, 26.5)	13.0 (6.0, 32.0)	7.00 (4.0, 14.0)

Abbreviation: IQR, interquartile range.

^a^Values are number (%) unless otherwise indicated. Percentages may not total 100 because of rounding.

^b^Infants include children attending and not attending kindergarten. Students include primary, secondary, and college students.

## Data Availability

The datasets used and analyzed during the current study are available from the corresponding author upon reasonable request.

## Supporting Information

Supporting Information Bayesian Spatiotemporal Model. Table S1. Characteristics of the variables included in the analysis. Table S2. Global spatial autocorrelation analysis of brucellosis in Yunnan, 2006–2021. Figure S1. Pearson correlation analysis of the factors influencing brucellosis in Yunnan, 2006–2021. Table S3. Factors influencing brucellosis based on Univariate Bayesian model for Yunnan, 2006–2021. Table S4. Comparison of DIC and WAIC for Bayesian spatiotemporal models.
