# Supplementary material for: Meteorological, Socioeconomic, and Environmental Factors Influencing Human Brucellosis Occurrence in Yunnan, China, 2006–2021: A Bayesian Spatiotemporal Modeling Study
Source: Transbound Emerg Dis. 2025 Jul 16;2025:8872434. doi: 10.1155/tbed/8872434 (PMC12286696; doi:10.1155/tbed/8872434)
Supplement: Supporting Information — Bayesian Spatiotemporal Model. Table S1. Characteristics of the variables included in the analysis. Table S2. Global spatial autocorrelation analysis of brucellosis in Yunnan, 2006–2021. Figure S1. Pearson correlation analysis of the factors influencing brucellosis in Yunnan, 2006–2021. Table S3. Factors influencing brucellosis based on Univariate Bayesian model for Yunnan, 2006–2021. Table S4. Comparison of DIC and WAIC for Bayesian spatiotemporal models. [file 8872434.f1.docx]

**Supplementary materials**

**Table of contents**

[Bayesian Spatiotemporal Model 2](#_Toc198146392)

[Table S1. Global spatial autocorrelation analysis of brucellosis in Yunnan, 2006–2021 3](#_Toc198146393)

[Table S2. Characteristics of the variables included in the analysis. 4](#_Toc198146394)

[Figure S1. Pearson correlation analysis of the factors influencing brucellosis in Yunnan, 2006–2021 5](#_Toc198146395)

[Table S3. Factors influencing brucellosis based on Univariate Bayesian model for Yunnan, 2006–2021 6](#_Toc198146396)

[Table S4. Comparison of DIC and WAIC for Bayesian spatiotemporal models 7](#_Toc198146397)

# Bayesian Spatiotemporal Model

The structured spatial effect at location $i{(\nu}_{i})$ was conditional on its neighbors $j\in\omega_{j}$ (where $\omega_{j}$ denotes the set of all adjacent neighbors of $i$). $n_{i}$ was the number of neighbors of $i$. $\tau_{1}$ and $\tau_{2}$ were the precision parameters.

$\nu_{i}|\nu_{-i}, \tau_{1}\sim Normal(\frac{1}{n_{i}}\sum_{j\in\omega_{j}} \nu_{j}, \frac{1}{n_{i}\tau_{1}})$ (1)

$\delta_{i}\sim Normal\left( 0,\left( \frac{1}{\tau_{2}} \right)^{2} \right)$ (2)

Temporal random effects $\mu_{t}$ were included to capture variability not explained by the model covariates. $\tau_{\mu}$ was the precision parameter.

$\gamma_{t}|\gamma_{t-1}\sim Normal(\gamma_{t-1},\sigma_{\gamma}^{2})$ (3)

$\mu_{t}\sim Normal(0,\frac{1}{\tau_{\mu}})$ (4)

Spatiotemporal interaction effect follows a normal distribution with precision ($\tau_{\varphi}$).

$\varphi_{st}\sim Normal(0,\frac{1}{\tau_{\varphi}})$ (5)

# Table S1. Global spatial autocorrelation analysis of brucellosis in Yunnan, 2006–2021

| Time | Moran’s *I* | Exp | Var | p | Z |
| --- | --- | --- | --- | --- | --- |
| 2006-2010 | 0.155 | -0.008 | 0.002 | <0.01 | 3.727 |
| 2011-2015 | 0.373 | -0.008 | 0.003 | <0.01 | 7.005 |
| 2016-2020 | 0.458 | -0.008 | 0.003 | <0.01 | 9.116 |
| 2021 | 0.438 | -0.008 | 0.002 | <0.01 | 9.892 |

# Table S2. Characteristics of the variables included in the analysis.

| Dataset | Variable | Data  period | Temporal  resolution | Spatial  resolution | Mean(Standard deviation)^1^ | Minimum–maximum |
| --- | --- | --- | --- | --- | --- | --- |
| NDVI | NDVI | 2006-2021 | Monthly | 1km | 0.87(0.07) | 0.32–1 |
| Land cover | Green coverage (%) | 2006-2021 | Yearly | 30m | 76.65(16.97) | 30.25–99.92 |
| DEM | Elevation (m) | 2000 | Yearly | 1km | 1844.72(502.8) | 753.83–3648.96 |
|  | Slope (°) | 2000 | Yearly | 1km | 5.51(2.44) | 1.46–13.22 |
| Water body | Distance to the nearest water body (m) | 2012 | Yearly | - | 245442.54(127313.61) | 627.61–511641.34 |
| Average temperature | Temperature (℃) | 2006-2021 | daily | monitoring  station | 17.08(5.08) | -3.98–26.28 |
| Average wind speed | Wind speed (m/s) | 2006-2021 | daily | monitoring  station | 1.81(0.45) | 0.65–3.64 |
| Precipitation | Precipitation（mm） | 2006-2021 | daily | monitoring  station | 80.35(74.69) | -2.14–415.96 |
| Nightlight | Nightlight (nW/cm2/sr) | 2006-2021 | Yearly | 500m | 0.33(1.18) | 0–14.48 |
| Population | Population (10,000 people) | 2006-2021 | Yearly | County | 35.6(23.47) | 3.4–161.09 |
| GDP | GDP (billion RMB) | 2006-2021 | Yearly | County | 891275.04(1446710.86) | 10100–14413700 |

^1^Variables were extracted and summarized to county and monthly scales first, and then descriptive statistics were calculated.

# Figure S1. Pearson correlation analysis of the factors influencing brucellosis in Yunnan, 2006–2021


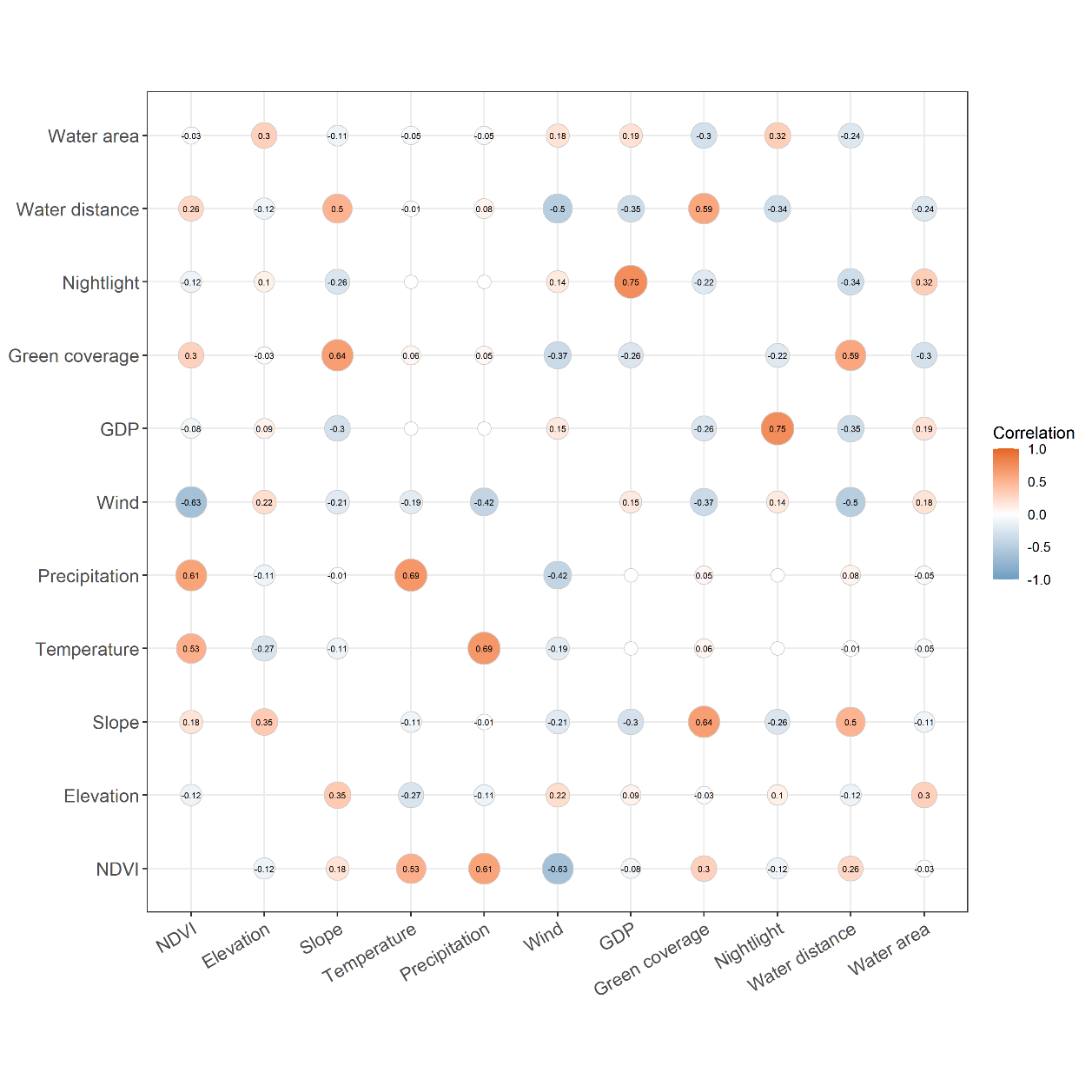


# Table S3. Factors influencing brucellosis based on Univariate Bayesian model for Yunnan, 2006–2021

| Variable | Coefficient | 95%BCI^¶^ |
| --- | --- | --- |
| Green coverage | -0.07 | (-0.074, -0.065) |
| Distance to the nearest water body | -0.01 | (-0.011, -0.009) |
| Slope | -0.474 | (-0.513, -0.435) |
| GDP | 0.008 | (0.007, 0.009) |
| Wind | 0.964 | (0.794, 1.135) |
| Elevation | 0.001 | (0.001, 0.001) |
| Water body area | 0 | (0.000,0.000) |
| Nightlight | 0.231 | (0.143, 0.32) |
| Precipitation | 0.003 | (0.002, 0.004) |
| Temperature | 0.033 | (0.018, 0.048) |
| NDVI | -1.161 | (-2.238, -0.083) |

^¶^ BCI, Bayesian credible interval

# Table S4. Comparison of DIC and WAIC for Bayesian spatiotemporal models

| Model | Model formula | DIC | WAIC |
| --- | --- | --- | --- |
| Model 1 | $\log\left( \rho_{st} \right)=\alpha+\sum\beta_{i}x_{ist}+\gamma_{t}+\mu_{t}$ | 10525 | 10525 |
| Model 2 | $\log\left( \rho_{st} \right)=\alpha+\sum\beta_{i}x_{ist}+\nu_{s}+\delta_{s}$ | 10481 | 10480 |
| Model 3 | $\log\left( \rho_{st} \right)=\alpha+\sum\beta_{i}x_{ist}+\gamma_{t}+\mu_{t}+\nu_{s}+\delta_{s}$ | 10463 | 10463 |
| Model 4 | $\log\left( \rho_{st} \right)=\alpha+\sum\beta_{i}x_{ist}+\gamma_{t}+\mu_{t}+\nu_{s}+\delta_{s}+\varphi_{st}$ | 10463 | 10462 |
